# Two New Compounds Based on Bi-Capped Keggin Polyoxoanions and Cu-Bpy Cations Contain Both Cu^II^ and Cu^I^ Complexes: Synthesis, Characterization and Properties

**DOI:** 10.3390/molecules28124706

**Published:** 2023-06-12

**Authors:** Yabing Liu, Wentong Zhao, Jijun Zheng, Huan Wang, Xiaobing Cui, Yaodan Chi

**Affiliations:** 1 Key Laboratory for Comprehensive Energy Saving of Cold Regions Architecture of Ministry of Education, Jilin Jianzhu University, Changchun 130118, China; wanghuan@jlju.edu.cn; 2 College of Material Science and Engineering, Jilin Jianzhu University, Changchun 130118, China; 3 State Key Laboratory of Inorganic Synthesis and Preparative Chemistry, College of Chemistry, Jilin University, Changchun 130021, China; cuixb@mail.jlu.edu.cn

**Keywords:** hydrothermal synthesis, crystallographic structure, polyoxometalates, catalysis, photocatalytic property

## Abstract

Two inorganic–organic hybrid complexes based on bi-capped Keggin-type cluster, {([Cu^II^(2,2′-bpy)_2_]_2_[PMo^VI^_8_V^V^_2_V^IV^_2_O_40_(V^IV^O)_2_])[Cu^I^(2,2′-bpy)]}∙2H_2_O (**1**) and {[Cu^II^(2,2′-bpy)_2_]_2_[SiMo^VI^_8.5_Mo^V^_2.5_V^IV^O_40_(V^IV^O)_2_]}[Cu^I^_0.5_(2,2′-bpy)(H_2_O)_0.5_] (**2**) (bpy = bipyridine), had been hydrothermally synthesized and structurally characterized by elemental analysis, FT-IR, TGA, PXRD and X-ray single-crystal diffraction analysis. Compound **1** consists of a novel 1-D chain structure constructed from [Cu^I^(2,2′-bpy)]^+^ unit linking bi-supported POMs anion {[Cu^II^(2,2′-bpy)_2_]_2_[PMo^VI^_8_V^V^_2_V^IV^_2_O_40_(V^IV^O)_2_]}^−^. Compound **2** is a bi-capped Keggin cluster bi-supported Cu-bpy complex. The main highlights of the two compounds are that Cu-bpy cations contain both Cu^I^ and Cu^II^ complexes. Furthermore, the fluorescence properties, the catalytic properties, and the photocatalytic performance of compounds **1** and **2** have been assessed, and the results show that both compounds are active for styrene epoxidation and degradation and adsorption of Methylene blue (MB), Rhodamine B (RhB) and mixed aqueous solutions.

## 1. Introduction

Owing to their unique topological versatility, electronic diversity, and rich physicochemical property, Polyoxometalates (POMs), a well-known kind of metal–oxygen cluster formed by groups VB and VIB transition metals (e.g., V^5+^, Nb^5+^, Mo^6+^ and W^6+^), have been used in the fields of catalysis, electromagnetism, sensors, medicine, nanomaterials [1,2,3,4,5,6,7,8,9,10,11,12,13,14,15,16,17,18]. Hybrid materials based on POMs have been extensively studied in coordination with chemistry and solid-state materials chemistry; since then, the first POMs were prepared in 1826 and the first structural details of Keggin POMs were reported in 1933 [19,20]. The number of POMs hybrids is also increasing exponentially with the improvement of characterization means and structural analysis techniques. In recent years, the design and synthesis of self-assembled POMs-based hybrid materials formed by combining POMs and transition metal complexes (TMCs) have turned out to be a hot topic in the field of POMs chemistry [21,22,23,24,25,26,27,28,29,30,31,32,33,34,35].

As one of the fundamental polyanions, the Keggin POMs anions have gained much attention, and a variety of structures composed of “substituted Keggin polyanions” [36,37,38,39,40,41], “capped Keggin polyanions” [42,43,44,45] and “substituted and capped Keggin polyanions” [46,47,48,49,50,51,52] have been reported, in which Keggin-type POMs anions are also regarded as the most desirable building blocks for the construction of inorganic–organic hybrid materials attributed to its intrinsic nonlinear optics, thermal stability, diverse electrons, redox properties, and so on [53,54,55,56]. Up to now, a new advancement has produced plenty of fascinating structures and better-performing hybrid materials passing through the intelligent selection between capped Keggin-type POMs and TMCs, and more researchers are devoted to the study of POM-TMC materials. TMCs are not limited to charging compensation but also can play a structure-directing role. The types of transition metals of TMCs include transition metal nickel, cobalt, silver, cadmium, zinc, copper, and so on. Among them, a variety of 1-D, 2-D, and 3-D extended structural POM-TMC including copper TMCs were reported because copper not only has two oxidation states of +2 and +1 but also has different coordination geometry [45,46,50,51,57,58,59,60]. However, to our knowledge, it is often ca single valence state of the Cu-based TMCs in previous reports, and copper in Cu-based TMCs provided with mixed valence states has rarely been reported [51,52,60].

Meanwhile, organic ligands that act as linkers and inducers to construct POM-TMC are more diverse in the choices used, especially for N- and O-containing ligands. Compared with flexible organic ligands, rigid ligands (e.g., bpy and phen) with conjugated systems that have strong coordination ability are more easily introduced into the chemical system to strengthen the stability of the skeletal structure of POMs. However, among the numerous experiments conducted with 2,2′-bpy as the organic ligand, the disordered 2,2′-bpy ligand acting as a linear structural ligand such as 4,4′-bpy connected into a one-dimensional chain structure with a novel POM-M-L-M-POM linking fashion has been only reported once in the literature [46].

Under the above background, we reported the preparation and characterization of two novel inorganic–organic hybrid materials based on bi-capped Keggin POMs and TMCs: {([Cu^II^(2,2′-bpy)_2_]_2_[PMo^VI^_8_V^V^_2_V^IV^_2_O_40_(V^IV^O)_2_])[Cu^I^(2,2′-bpy)]}∙2H_2_O (**1**) and {[Cu^II^(2,2′-bpy)_2_]_2_[SiMo^VI^_8.5_Mo^V^_2.5_V^IV^O_40_(V^IV^O)_2_]}[Cu^I^_0.5_(2,2′-bpy)(H_2_O)_0.5_] (**2**), in which compound **1** consists of a 1-D chain structure and compound **2** is a discrete structure. The main highlights of the two compounds are that Cu-bpy cations contain both Cu^I^ and Cu^II^ complex. Moreover, compounds **1** and **2** exhibit transcendent fluorescence properties, catalytic performance in the epoxidation of styrene, and the capability of the photodegradation of organic dyes.

## 2. Results and Discussion

### 2.1. Synthesis Discussion

Compound **1** was isolated from the reaction of Na_3_[P(Mo_3_O_10_)_4_]·xH_2_O, V_2_O_5_, CuCl_2_·2H_2_O, H_2_C_2_O_4_·2H_2_O, 2,2′-bpy and distilled water at 180 °C for 3 days by the hydrothermal method with pH adjusted to 4, while compound **2** was separated from the hydrothermal reaction of Na_2_MoO_4_, Na_2_SiO_3_·9H_2_O, V_2_O_5_, CuCl_2_·2H_2_O, H_2_C_2_O_4_·2H_2_O, 2,2′-bpy and distilled water in similar conditions to compound **1**. In the synthesis reactions, H_2_C_2_O_4_·2H_2_O does not appear to be involved in the play and part in the assembly of compounds **1** and **2**. To study the effects of H_2_C_2_O_4_·2H_2_O, we tried to synthesize **1** and **2** under the same conditions without H_2_C_2_O_4_·2H_2_O, but no desired crystal was found. It means that H_2_C_2_O_4_·2H_2_O not only influences the pH values of the system but also acts as a reducing agent under hydrothermal conditions. Mo^VI^, V^V^ and Cu^II^ in the starting materials of **1** and **2** were reduced to Mo^V^, V^IV^ and Cu^I^ in the presence of H_2_C_2_O_4_·2H_2_O.

### 2.2. Description of Crystal Structures

#### 2.2.1. {([Cu^II^(2,2′-bpy)_2_]_2_[PMo^VI^_8_V^V^_2_V^IV^_2_O_40_(V^IV^O)_2_])[Cu^I^(2,2′-bpy)]}∙2H_2_O (**1**)

The asymmetric unit of compound **1** is made up of half a bi-capped pseudo-Keggin cluster anion [PMo_8_V_6_O_42_]^5−^, one [Cu(2,2′-bpy)_2_]^2+^ cation, half a [Cu(2,2′-bpy)]^+^ cation, and two crystalline water molecules. The polyoxoanion [PMo_8_V_6_O_42_]^5−^ can be described as the pseudo-Keggin molybdenum–vanadium cluster [PMo_8_V_4_O_40_]^9−^ with two additional [VO]^2+^ caps on its two opposite pits. The disordered [PO_4_]^3−^ is close to a cubic configuration which is formed from two half-occupied tetrahedral encapsulated {PO_4_} with a P-O bond length between 1.504(8)–1.578(8) Å and O-P-O bond angles ranging from 105.7(4)–112.5(5) Å. All molybdenum centers have a distorted {MoO_6_} octahedral environment, and all vanadium exhibit a {VO_5_} square pyramidal environment. The M-O (M = Mo and V) bond lengths fall into three types according to the different coordination environments: M-O(t), M-O(b) and M-O(c), and O(t), O(b) and O(c) are elaborated terminal oxygen, bridging oxygen and central oxygen atoms, respectively. The bond lengths are 1.659(5)–1.667(5) Å for Mo-O(t) and 1.590(5)–1.658(5) Å for V-O(t); 1.786(6)–2.066(5) Å for Mo-O(b) and 1.909(7)–1.967(7) Å for V-O(t); the bond lengths of 2.428(9)–2.494(8) Å for Mo-O(c) and 2.460(9) Å for V-O(c). [PMo_8_V_6_O_42_]^5−^ is similar to that of [PMo_12_V_2_O_42_]^5−^ in that both consists of the well-known Keggin cluster anion with two five-coordinated [VO]^2+^ caps attached, but differs in that the four {MoO_6_} octahedra in [PMo_12_V_2_O_40_]^5−^ were replaced by the four {VO_5_} tetragonal vertebrae, forming a substituted Keggin-type cluster anion with [PO_4_]^3−^ centered vanadium-oxygen and molybdenum-oxygen arranged in {VO_4_}/{Mo_4_O_18_}/4{VO_5_}/{Mo_4_O_18_}/{VO_4_} lamellar rules (Supporting Information, Figure S1). Applying the empirical BVS (bond valence sum) calculations formula *S =* exp[−(*R_1_* − 1.88)/0.37] (*S* = bond value, *R*_1_ = Mo-O bond length), the calculated *S* values of Mo1–Mo4 are 5.97, 6.00, 6.01 and 6.12, respectively, indicating that the oxidation state of all the Mo atoms is +6; applying *S =* exp[−(*R_2_* − 1.790)/0.319](*R*_2_ = V-O bond length), the *S* values of V1–V3 are 4.42, 4.40 and 4.21, respectively, indicating that the oxidation state of two V atoms is +5 and four is +4 [61]. Therefore, the bi-capped pseudo-Keggin cluster anion is expressed as [PMo^VI^_8_V^V^_2_V^IV^_2_O_40_(V^IV^O)_2_]^5−^.

**Figure 1:**
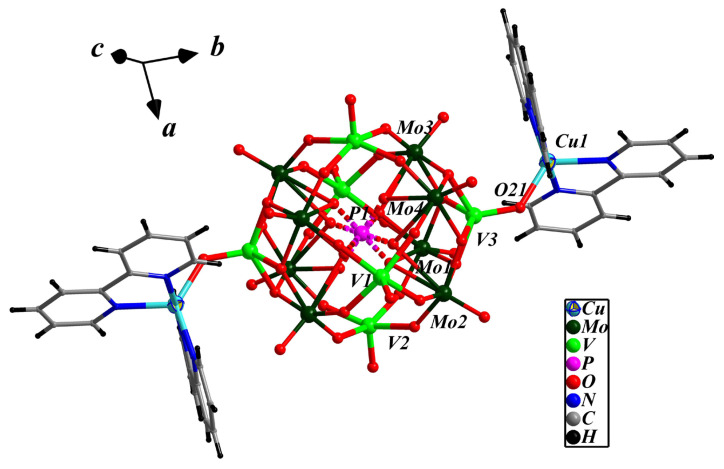
The ball-and-stick diagram of the POM supported coordination in compound **1**.

**Figure 2:**
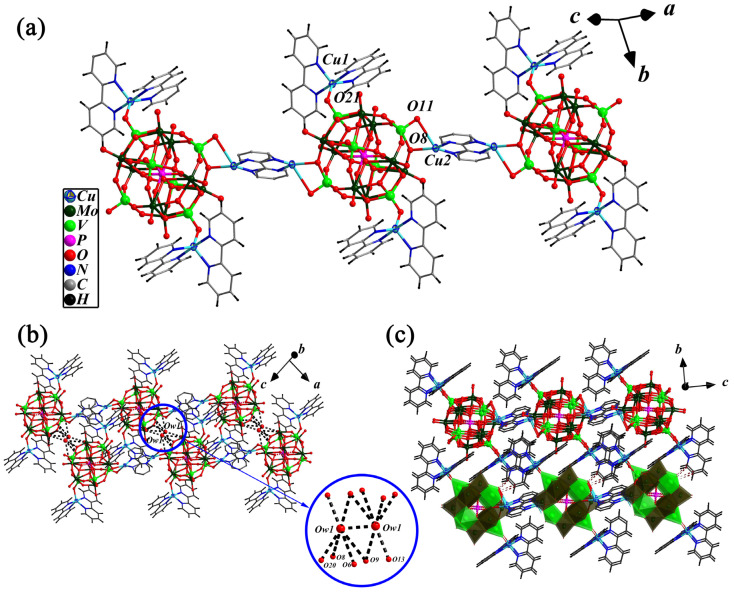
Ball-and-stick representation of the 1-D chain (**a**), the 2-D supramolecular layer structure formed through the Ow…O hydrogen bonds in (H_2_O)_2_ cluster and two neighboring 1-D chains (**b**), and the view of 3-D hydrogen-bonded framework with the C-H…O and N-H…O hydrogen bond interactions between two 2-D layers along the *bc* plane (**c**) in compound **1**.

There are two Cu-bpy TMCs in compound **1**: [Cu(1)(2,2′-bpy)_2_]^2+^ and [Cu(2)(2,2′-bpy)]^+^. Cu(1) and Cu(1a) (symmetry codes: 1 − *x*, −*y*, 2 − *z*) of [Cu(1)(2,2′-bpy)_2_]^2+^ coordinate with four N donors from two 2,2′-bpy molecules, respectively, forming [Cu(1)(2,2′-bpy)_2_]^2+^ cations with the Cu(1)-N bond distances of 1.964(6)–2.086(7) Å. As shown in Figure 1, the two Cu(1) complex cations interacts with two terminal oxygen O(21) and O(21a) of the cluster anion [PMo_8_V_6_O_42_]^5−^ to form the bi-supported complex anion {[Cu(1)(2,2′-bpy)_2_]_2_[PMo_8_V_6_O_42_]}^−^ with the bond length of 2.005(5) Å for Cu(1)-O(2). [Cu(2)(2,2′-bpy)]^+^ is a linker, in which two half-occupied Cu(2) and Cu(2a) (symmetry codes: 1 − *x*, −*y*, 2 − *z*) receives contributions from four half-occupied N donors of a disordered 2,2′-bpy molecule (Figure S2), a terminal oxygen O(11) and a bridging oxygen O(8) of the bi-supported complex anion {[Cu(1)(2,2′-bpy)_2_]_2_[PMo_8_V_6_O_42_]}^−^ to form a novel -POM-Cu(2)-2,2′-bpy-Cu(2a)-POM- special type one-dimensional chain structure (Figure 2a). The Cu(2)-N distances are 2.011(15) and 2.090(3) Å, and the Cu(2)-O distances are 2.194(10) and 2.787(4) Å respectively. A review of the literature revealed that a disordered 2,2′-bpy ligand acting as a linear structural ligand linked into a 1-D chain structure has been rarely reported [46].

A highlighting feature of compound **1** is that two Ow1 form an (H_2_O)_2_ water cluster with O…O distances of 2.7053(2) Å which is connected to the adjacent 1-D chain to 2-D supramolecular layers. As shown in Figure 2b, Ow1 of (H_2_O)_2_ cluster interactions with O8(#1), O9, O13, O20(#2) and O9(#1) from two {([Cu^II^(2,2′-bpy)_2_]_2_[PMo^VI^_8_V^V^_2_V^IV^_2_O_40_(V^IV^O)_2_])[Cu^I^(2,2′-bpy)]}_∞_ chains through Ow1…O hydrogen bonds with a bond length range of 2.849(2)–3.337(2) Å. Meanwhile, along the *bc* plane, the adjacent layers interacted with each other via C-H...O and N-H...O hydrogen bonds to form another 3-D supramolecular network with the bond length range of 3.034(2)–3.384(2) Å (Figure 2c). The typical Ow…O, Ow…Ow, C-H…O and N-H…O hydrogen bonds forming the 3-D supramolecular structure of this compound **1** are given in Table 1.

One analogous compound [{Cu^II^(bpy)}{Cu^II^(bpy)_2_}_2_(V^IV^O)_2_(AsMo^VI^Mo^V^_7_V^V^_4_O_40_)]·H_2_O (**3**) has been reported by Yu et al. in 2021 [46], but there exist major differences between **1** and **3**. Firstly, bi-capped Keggin polyoxoanion in **3** is not [PMo^VI^_8_V^V^_2_V^IV^_2_O_40_(V^IV^O)_2_]^5−^, but [AsMo^VI^Mo^V^_7_V^V^_4_V_2_^IV^O_42_]^6−^, and in both the central atom and valence are all discrepant. Secondly, the π…π interactions are a critical and non-negligible element in the formation of the packing structure in **3**, but no strong π…π interactions exist in **1**. The hydrogen bonds of (H_2_O)_2_ water cluster and POMs increase the stability of the crystal of **1**. Thirdly, disordered 2,2′-bpy in **3** is C/N co-occupying sites with the ratio of 0.5, and the C and N latter of 2,2′-bpy in **1** is disorderedly occupied over four positions (N5 and C21, N6 and C22) with the occupancy of 0.5 (Figure S2). Last and most important, all the copper in the Cu-bpy cations of **3** are in the +2 valence, but the copper cations contain both +2 and +1 valence in **1**.

#### 2.2.2. {[Cu(2,2′-bpy)_2_]_2_[SiMo^VI^_8.5_Mo^V^_2.5_V^IV^_3_O_42_]}[Cu_0.5_(2,2′-bpy)(H_2_O)_0.5_] (**2**)

**Figure 3:**
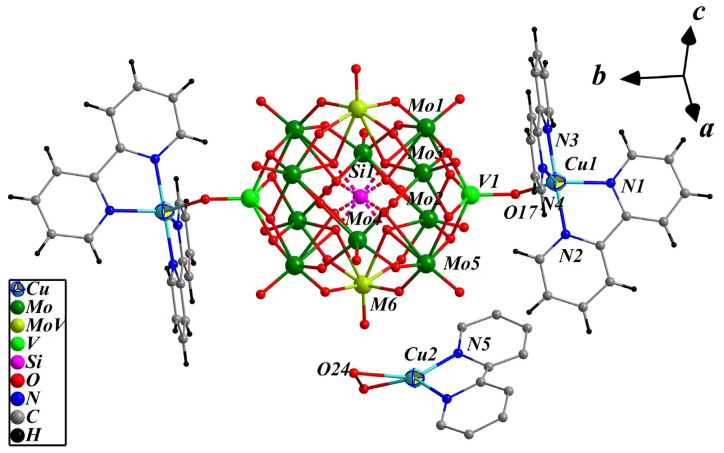
The molecular structure of {[Cu^II^(2,2′-bpy)_2_]_2_[SiMo_11_V_3_O_42_]}[Cu^I^_0.5_(2,2′-bpy) (H_2_O)_0.5_].

Compound **2** belongs to the *C*2/c spatial group of monoclinic and the basic structural unit is composed of a bi-capped pseudo-Keggin cluster anion [SiMo_11_V_3_O_42_]^3−^, two Cu^II^ complex cation [Cu(2,2′-bpy)_2_]^2+^ and Cu^I^ complex cation [Cu_0.5_(2,2′-bpy)(H_2_O)_0.5_]^0.5+^ (Figure 3). The cluster anion [SiMo_11_V_3_O_42_]^4.5−^ in compound **2** extremely resembled [PMo_8_V_6_O_42_]^5−^ in compound **1**, except phosphorus is replaced by silicon and the quantities of molybdenum and vanadium differed, and the major variance of them is that there are two Mo/V co-occupying sites with the ration of Mo: V = 0.5:0.5 in **2**. Disordered {SiO_4_} cube is present at the center of the cluster anion with a Si-O bond length range of 1.617(7)–1.649(8) Å. Ten {MoO_6_} and two {MO_6_} (M = 0.5Mo + 0.5V) distorted octahedra form two {Mo_3_O_13_} groups and two {Mo_2.5_V_0.5_O_13_} groups surround the two half-occupied {SiO_4_} tetrahedra to form mono-V substituted pseudo-Keggin POMs. The bond length ranges of Mo-O(t), Mo-O(b) and Mo-O(c) are 1.627(6)–1.678(5), 1.914(7)–1.945(8) and 2.406(8)–2.427(8) Å; the disordered M-O(t), M-O(b) and M-O(c) bond distances are 1.622(6), 1.911(8)–1.946(9) and 2.417(8) Å; the bond lengths of V-O(t) and V-O(b) are 1.640(5) and 1.910(6)–1.925(6) Å, respectively, which are similar to the structural data reported in the literature [62]. Applying the same empirical BVS calculations formula as **1**, the Mo1–Mo6 valences are 5.83, 5.78, 5.68, 5.81, 5.77 and 5.76, and the total valence sum of all Mo atoms is 63.50, which determines that the eleven molybdenum consisting of 8.5 are in +6 and 2.5 are in +5; the valence of V1 is 4.14 and the valence of the disordered V6 is 4.21, which reveals that all three V are in +4 oxidation states. The structural formula of the cluster anion [SiMo_11_V_3_O_42_] was thus determined to be [SiMo^VI^_8.5_Mo^V^_2.5_V^IV^O_40_(V^IV^O)_2_]^4.5−^.

The transition metal coordination copper ions in **2** comprise two types of [Cu(1)(2,2′-bpy)_2_]^2+^ and [Cu(2)_0.5_(2,2′-bpy)(H_2_O)_0.5_]^0.5+^. The Cu(1) cation exhibits a five-coordination configuration, which associates with four nitrogen atoms from two 2,2′-bpy ligands and one terminal oxygen atom from [SiMo_11_V_3_O_42_]^4.5−^; Cu(1)-N bond lengths range from 1.968(6) to 2.071(6) Å. From another standpoint, both O17 and O(17a) atoms (symmetry codes: 0.5 − *x*, 0.5 − *y*, 1 − *z*) on the two {VO} caps of [SiMo_11_V_3_O_42_]^4.5−^ serve as bridging ligands coordinating to two copper cations from [Cu(1)(2,2′-bpy)_2_]^2+^ that form a bi-supported {[Cu(1)(2,2′-bpy)_2_]_2_[SiMo_11_V_3_O_42_]}^0.5−^ structure, with a Cu(1)-O(17) distance of 2.020(3) Å. Coordination patterns of Cu(2) ions are very rare. The half-occupancy Cu(2) cation is coordinated with the two nitrogen atoms of one 2,2′-bpy ligand and a semi-water molecule composed of two disorder one-quarter water molecules (O24 and O24′, symmetry codes: 1 − *x,y*,0.5 − *z*) whose O…O bond length is 1.600(1) Å, forming a counter cation [Cu(2)_0.5_(2,2′-bpy)(H_2_O)_0.5_]^0.5+^. The bond lengths of Cu(2)-N and Cu(2)-O are 1.976(9) and 2.244(4) Å, respectively.

**Figure 4:**
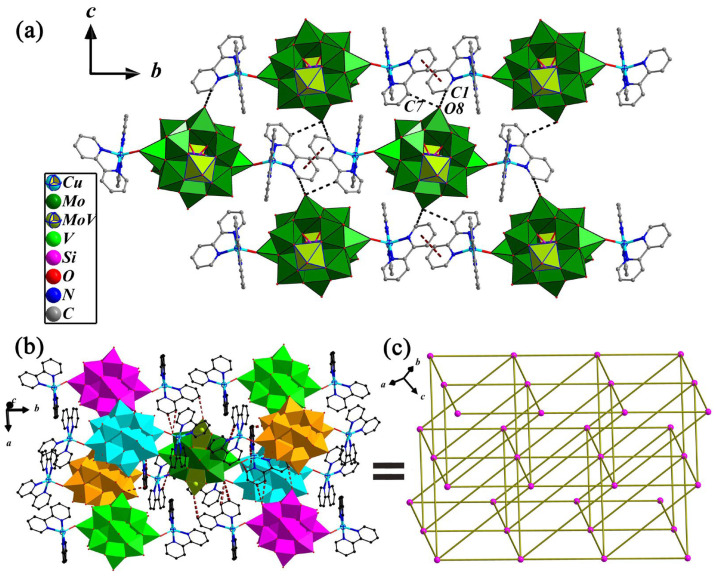
(**a**) View of the 1-D chain and 2-D layer structures with the π…π interactions and C-H…O hydrogen bond interactions in **2**; several hydrogens are omitted for clarity. (**b**) Combined ball-and-stick and polyhedron representation of {[Cu(1)(2,2′-bpy)_2_]_2_[SiMo_11_V_3_O_42_]}^0.5−^ anion connected to eight adjacent anions through C-H…O and N-H…O hydrogen bonding interactions on the *ac* plane. (**c**) Schematic drawing of the 3-D supramolecular topology in **2**.

The structural novelty of compound **2** lies in the presence of C-H…O, N-H…O hydrogen bond and the π…π stacking between the 2,2′-bpy aromatic rings, which makes the structure of compound **2** novel and stable. As shown in Figure 4a, there exists only a kind of π…π stacking interaction that occurs between identical N1 pyridine rings that come from two adjacent bi-supported POMs to form an infinite 1-D supramolecular chain with the centroid–centroid distance of two rings being 3.708 Å. Two adjacent supramolecular chains are connected via C-H…O hydrogen bonds to form a 2-D supramolecular layered structure. The schematic representation of the 3-D network for **2** that built up the bi-supported {[Cu(1)(2,2′-bpy)_2_]_2_[SiMo_11_V_3_O_42_]}^0.5−^ through C-H…O hydrogen bonds is delineated in Figure 4b. Being a building block, each bi-supported POMs anion linked with eight adjacent POMs anions assembles into a 3-D supramolecular network, and the 3-D supramolecular topological structure can be obtained in **2** if the bi-supported {[Cu(1)(2,2′-bpy)_2_]_2_[SiMo_11_V_3_O_42_]}^0.5−^ is perceived as nodes and hydrogen bonds as spacers (shown in Figure 4c). The selected hydrogen bonds are also listed in Table 1.

Using the BVS calculation formula *S =* exp[−(*R*_3_ *−* 1.709)/0.37] (*S* = bond value, *R*_3_ = Cu-N bond length) and *S =* exp[−(*R*_4_ − 1.679)/0.37] (*S* = bond value, *R*_4_ = Cu-O bond length) by Brown, the oxidation state of the copper was given that Cu(1) is 2.11 and Cu(2) is 1.04 for **1**, and Cu(1) is 2.12 and Cu(2) is 0.92 for **2**, respectively. Their results coincided with the charge balance and indicated that the Cu-bpy TMCs of **1** and **2** are mixed valence states, which has been rarely reported in previous literatures [51,52,60].

### 2.3. FT-IR Spectrophotometry

The FT-IR spectra of compounds **1** and **2** were recorded in the regions between 4000 and 400 cm^−1^ and exhibit the characteristic peaks of Keggin POMs (Figure S3). The characteristic peaks located at 945, 914, 773 cm^−1^ for **1** and 949, 794, 770 cm^−1^ for **2** are ascribed to the stretching vibration of *ν*(M-O(t)), *ν*(M-O(b)), and *ν*(M-O(c)) (M = Mo or V), respectively. The peaks at 1048 cm^−1^ for **1** and 891 cm^−1^ for **2** are connected with the *ν*(P-O) vibrations of {PO_4_} and *ν*(Si-O) vibrations of {SiO_4_}. The peaks in the range of 1608–1166 cm^−1^ for **1** and 1658–1165 cm^−1^ for **2** can be ascribed to *ν*(C=N) and *ν*(C=C) vibrations of 2,2′-bpy ligands. The characteristic peak at 3438 cm^−1^ for **1** is connected with the *ν*(O-H) vibration in lattice water molecules.

### 2.4. X-ray Photoelectron Spectroscopy

The XPS spectra of the molybdenum of compounds **1** and **2** are shown in Figure S4. Figure S4a gives two peaks at 236.0 and 232.9 eV for **1**, attributed to Mo^6+^ 3d_3/2_ and Mo^6+^ 3d_5/2_, and Figure S4b shows four overlapped peaks at 236.1, 234.5, 232.7 and 231.4 eV for **2**, attributed to Mo^6+^ 3d_3/2_, Mo^5+^ 3d_3/2_, Mo^6+^ 3d_5/2_ and Mo^5+^ 3d_5/2_, respectively, indicating that the oxidation states of molybdenum are +6 in compound **1,** and are the mixture of +5 and +6 in compound **2**. The XPS spectra of vanadium in compounds **1** and **2** are shown in Figure S5. The XPS spectrum of vanadium in compound **1** gives four overlapped peaks at 524.5, 523.3, 516.9 and 515.8 eV, attributed to V^5+^ 2p_3/2_, V^4+^ 2p_3/2_, V^5+^ 2p_1/2_ and V^4+^ 2p_1/2_, respectively, corresponding to the oxidation states of vanadium in compound **1** being +4 and +5 (Figure S5a). The XPS spectrum of vanadium in compound **2** is different from compound **1**, which exhibits two peaks at 516.2 and 523.9 eV, we assigned these peaks to V^4+^ in compound **2** (Figure S5b). The XPS analyses are in good accordance with the BVS.

### 2.5. Thermogravimetric Analyses

The thermogravimetric analyses (TGA) curves for **1** and **2** between 40 °C and 900 °C under the N_2_ atmosphere are presented in Figure S6. The TG curve for **1** exhibits a two-stage weight loss. The first stage lost 1.27% of weight in 40–210 °C (calcd. 1.29%), which is consistent with the vaporization of two water molecules. The next stage loss is 27.98% (calcd. 28.01%) from 338 to 675 °C, which can be attributed to the removal of five 2,2′-bpy ligands. The TG curve for **2** exhibiting one-stage weight loss of 27.67% (calcd. 28.23%) from 248 to 854 °C can be caused by the removal of five 2,2′-bpy ligands and one-half of a water molecule. The total weight loss for compound **1** is 29.25%, which is consistent with the calculated value of about 29.30%, attributed to the release of five 2,2′-bpy and two water molecules. On the other hand, the total weight loss for compound **2** is 27.67%, which is consistent with the calculated value of about 28.23%, attributed to the release of five 2,2′-bpy and a semi-water molecule.

### 2.6. Powder X-ray Diffraction

The powder X-ray diffractions (PXRD) were studied at an angle range of 0–50° to analyze the purity of the sample of the two title compounds. The experimental PXRD patterns of compounds **1** and **2** are in great agreement with the simulated ones, and the intensity of the peaks are slightly different, indicating the crystal phase purity, as shown in Figure S7. The preferred orientations of crystalline samples for compounds **1** and **2** eventually led to the distinction in reflection intensity.

### 2.7. UV-vis Spectrophotometry

The UV-vis spectra for compounds **1** and **2** were measured in the range of 200–800 nm and are revealed in Figure S8. The UV-vis spectra have shown an intense absorption band at 255 nm and a broad band at 284 nm of **1**, a sharp absorption peak at 253 nm and a broad peak at 286 nm of **2**. Both of them are all due to pπ(O(t)/O(b)/O(c))-dπ* (Mo/V) nuclear transitions in Mo/V-O bonds [31,63]. 

### 2.8. Fluorescence Properties

Aromatic organic ligands with conjugated structures have special fluorescence properties, so organic–inorganic hybrid materials with aromatic ligands in their structures will also have salutary fluorescence properties, which have been practical applications in numerous fields, such as chemical sensing and photoluminescence. The fluorescent properties of the free 2,2′-bpy and two title compounds were examined in the solid state at room temperature, and the emission spectra are revealed in Figure S9. The fluorescent spectrum of 2,2′-bpy displays an emission peak at 415 nm (λ_ex_ = 366 nm). The fluorescent spectra of compounds **1** and **2** exhibit similar emission peaks at 423 nm (λ_ex_ = 372 nm) and 421 nm (λ_ex_ = 376 nm), respectively. It is clear that the emission bands of compounds **1** and **2** are similar to a free 2,2′-bpy ligand in terms of the position and band shape, and these bands should be assigned to the intra-ligand charge transition of 2,2′-bpy. 

### 2.9. Catalytic Properties

Using an aqueous solution of tert-butyl hydrogen peroxide (TBHP) as a strong oxidant and compounds **1** and **2** as the catalyst, the epoxidation of styrene to styrene oxide with TBHP was carried in a batch reactor which was probed by the catalytic performances of **1** and **2**. 2 mg (0.72 µmol) of finely ground compounds **1** and **2**; 0.114 mL (1 µmol) of styrene and 2 mL CH_3_CN were dropped to a 10 mL double-necked flask equipped with a reflux condenser and stirrer. The mixture solution was heated to 80 °C in an oil bath and then 2 mL of TBHP was injected to start the reaction which lasted 8 h. A gas chromatograph (Shimadzu, GC-8A) with a flame detector and an HP-5 capillary column were used to quantify the organic composition of the reaction system, and the catalytic activity of compounds **1** and **2** was evaluated based on the conversion of styrene and product selectivity. The same conditions were tested for zero styrene conversion in the absence of the catalyst.

**Figure 5:**
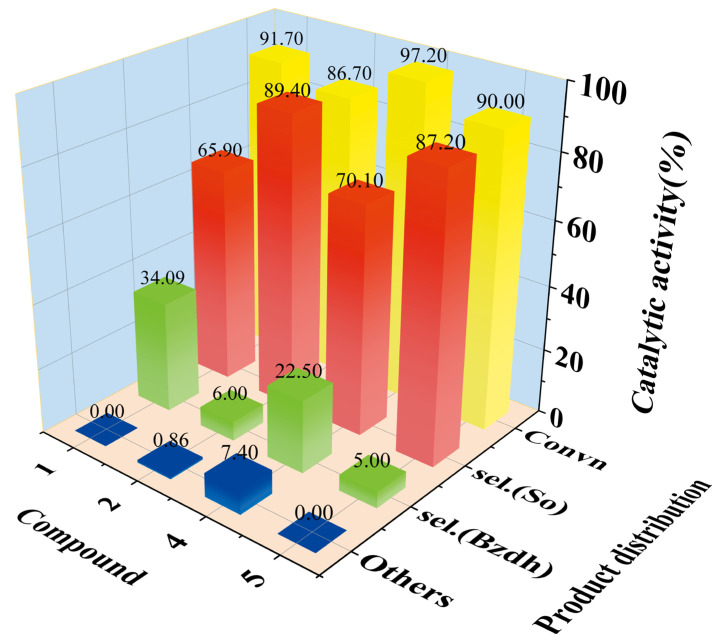
Catalytic activity and product distribution of compounds **1**, **2**, **4** and **5**. (Convn = Styrene conversion; So = styrene oxide; Bzdh = benzaldehyde; others: including benzoic acid and phenylacetaldehyde).

The results of the catalytic reactions for styrene oxidation by TBHP using various compounds as catalysts are presented in Figure 5 and more data information is in Table S1. It is of note that both **1** and **2** presented activity for styrene oxidation. As a catalyst, the total conversions of compounds **1** and **2** were 91.7% and 86.7% after 8 h, and the selectivity of the main target products, phenylethylene oxide: yard, was 65.9% and 89.4%, respectively. The excellent catalytic activity of compounds **1** and **2** is attributed to the novel structure including Keggin Mo-V polyoxoanions and Cu-bpy cations containing both Cu^II^ and Cu^I^ complexes, which are consistent with Cui et al., who demonstrated the type and structure of catalysts with high catalytic activity and stability in styrene oxidation, such as [PMo_10_V_4_O_42_][Cu^I^_2_(4,4′-bpy)_2_][Cu^II^(phen)_2_]_2_ (**4**) and [PMo_8_V_6_O_42_][Cu^II^(2,2′-bpy)_2_]_2_[Cu^I^(2,2′-bpy)]·3H_2_O (**5**) [51,52].

The recyclability and reusability of compounds **1** and **2** were also studied, including the conversion and catalyst recovery in three cycles. The results are shown in Table 2. The same experimental conditions were employed except that 10 mg of compounds **1** and **2** were used as catalysts. Because of the increase in the dose of compounds **1** and **2**, the conversion increased to 93.0% for **1** and 87.3% for **2**, and the selectivity increased to 72.3% for **1** and 91.1% for **2**. The catalysts were recovered by filtration and washed with acetonitrile when every cycle was over. After they were dried at room temperature, the recovered compounds **1** and **2** were directly reused. The catalytic activity of compounds **1** and **2** did not exhibit a significant decrease even over three cycles, and the conversions of compounds **1** and **2** from the first to three cycles are 93.0%, 90.5% and 92.6% for **1**, and 87.3%, 84.9%, 83.8% for **2,** respectively. The selectivity of compounds **1** and **2** from the first to three cycles are 72.3%, 60.8% and 75.2% for **1**, and 91.1%, 85.8% and 91.5% for **2**, respectively. The residual catalysts of compounds **1** and **2** were recorded in the power XRD patterns and IR spectra to compare whether the structures changed. The IR spectra and the power XRD patterns of compounds **1** and **2** after three cycles are shown in Figures S10 and S11, and the main characteristic peaks of suspended solids after three runs of repeated experiments of compounds **1** and **2** have little change, which are still consistent with experimental ones. It means that the samples have good stability in catalytic experiments.

### 2.10. Photocatalytic Activities

Safely and effectively disposing of industrial wastewater is one of the most challenging problems in environmental governance [64,65,66,67]. Typical organic dyes such as RhB and MB were chosen to simulate organic pollutants, and the reaction process of the system was monitored by the change of absorbance intensity at the characteristic absorption wavelength of different organic dyes to judge its ability to decontaminate industrial wastewater and further evaluate the photocatalytic activity of cluster-based hybrid materials **1** and **2**. The experimental procedure is as follows: (a) 5 mg of fine powder of compounds **1** and **2** were sufficiently ground in an agate mortar and dissolved in 200 mL aqueous solutions of RhB (1.0 × 10^–5^ mol∙L^–1^), the reaction system was adjusted to pH = 1, 3 and 10, and the catalyst was dispersed uniformly by ultrasonic shaking to form a suspension. (b) The suspension was stirred on a magnetic stirrer about 20 min in the dark environment, to achieve a adsorption–desorption balance between the catalyst and RhB aqueous solution. A total of 5 mL suspension with solids was removed by centrifugal force at 5000 rpm, and the liquid supernatant was tested by UV-vis spectroscopy and the absorbance value was measured and recorded. (c) The reaction system was irradiated with a 400 W Xe lamp, and the liquid was centrifuged at 10,000 rpm at 5 cm from the lamp. A 5 mL sample was analyzed every 20 min by UV-vis spectroscopy. As a photocatalyst of compounds **1** and **2**, the UV-vis absorption curves of **1** and **2** for photocatalytic degradation RhB solution with different reaction time and pH values were presented in Figures S12a–c and S13a, and the concentration of RhB solution (*C_t_*/*C*_0_) versus irradiation time was plotted in Figures S12d and S13d. After 120 min reaction, the degradation rates of the RhB solution at pH = 1, 3 and 10 were 74.1%, 85.8%, 79.5% for **1**, and 93.9%, 94.0%, 83.8% for **2**, respectively. Compounds **1** and **2** present better activity for the degradation of RhB at pH = 3.

**Figure 6:**
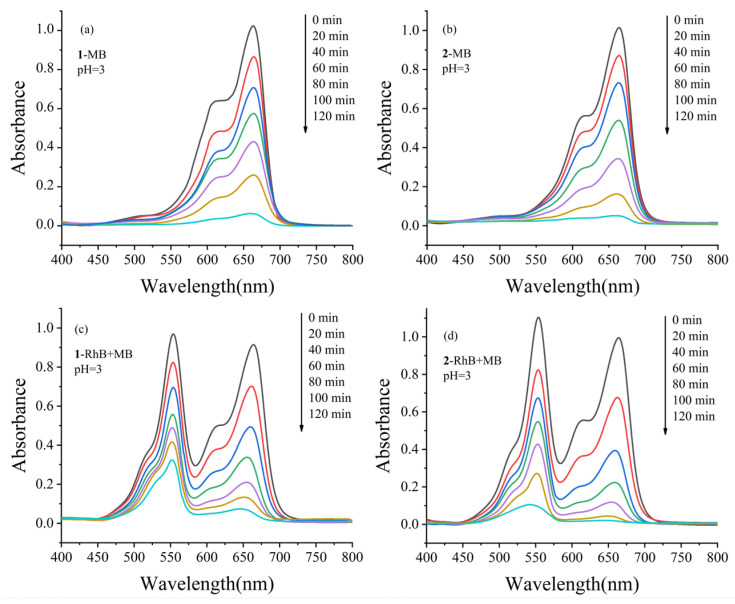
Absorption spectra of the MB and MB + RhB solution under UV-vis irradiation with the presence of compounds **1** and **2** at pH = 3. (**a**) **1**-MB, (**b**) **2**-MB, (**c**) **1**-RhB + MB and (**d**) **2**-RhB + MB. The black, red, blue, green, purple, and light blue lines represent the absorption spectra curves for tests performed at 0, 20, 40, 60, 80, 100, and 120 min, respectively.

The same operation method was used and the pH of the reaction system was adjusted to about 3 to test the photocatalytic performances for MB and RhB + MB aqueous solution. The UV-vis absorption curves of compounds **1** and **2** with reaction time for the photocatalytic degradation MB and MB + RhB solution were revealed in Figure 6, and changes in the concentration of MB solution (*C_t_*/*C*_0_) versus irradiation time were shown in Figure S14. After a 120 min reaction, the degradation rates of MB in only one MB dye solution reached 93.9% for **1** and 95.1% for **2** (Figure 6a,b). The degradation rates of RhB and MB were 66.6% and 92.1% for **1** (Figure 6c), 90.4% and 98.0% for **2** (Figure 6d) in a mixed solution containing MB and RhB. The results showed that compounds **1** and **2** both had a superior photocatalytic performance for the degradation of RhB, MB in MB + RhB solution. It is noteworthy that both compounds **1** and **2** showed slightly smaller catalytic degradation of RhB and MB in mixed solution than in pure solution, and the reasons for these phenomena need to be further investigated.

Table S2 shows the reaction results of the photodegradation of RhB by different catalysts in the literature [68,69,70]. [NBu_4_]_4_[SiW_12_O_40_] (**6**) based on [NBu_4_]^+^ and tungstate-based Keggin species; [Cu_2_(bpy)][H_2_SiW_12_O_40_]·(bib)·2H_2_O (**7**) and compound **1** have similar MOFs, but different POMs; [Zn(bix)_4_][PMo_9_V_3_O_40_(VO)_2_]·2H_2_O (**9**), [HMn(bix)_4_][PMo_8_V_4_O_40_(VO)_2_]·2H_2_O (10) and [Cu(bix)_4_][PMo_9_V_3_O_40_(VO)_2_]·4H_2_O (**11**) are similar to compound **1** POMs, but different from MOFs and organic ligands; [Cu_2_(bipy)][H_2_SiW_12_O_40_]·(bib)·2H_2_O (**7**) and {[Co_2_(btp)_3_(H_2_O)_6_](α-SiW_12_O_40_)·3H_2_O}_n_ (**8**) have similar POMs, but different from metal complexes. A comprehensive analysis shows that as a POMs-based photocatalyst, the factors affecting the catalytic ability of POMs-based hybrids for the degradation of organic pollutants are as follows: (1) the types and structures of polyoxoanions. Compounds **9**, **10** and **11** were based on Mo-V-O polyoxoanions, and the activities are higher than compounds 7 and 8 which constituted W-O polyoxoanions; (2) contribution of transition metal ions. Fixing POMs on porous MOF structures with different transition metals (Cu^2+^, Cu^+^, Co^2+^, Zn^+^ and Mn^2+^) can increase the surface area while maintaining their excellent catalytic activities [71].

## 3. Experimental Section

### 3.1. Materials and Methods

All the chemical reagents are analytical pure from commercially purchased sources and have not been further purified for the experiment. The elemental analyses (C, H and N) were recorded on a Multi EA 5100 elemental analyzer (Jena, Germany) and Cu, Mo, V, P and Si elemental analyses were performed on a Perkin-Elmer Optima 7300 V spectrophotometer (Waltham, MA, USA). Fourier-transform infrared (FT-IR) spectra for the solid samples were measured (Bruker VERTEX 70v, Billerica, MA, USA) in the range of 400–4000 cm^−1^ by pressing KBr pellets. X-ray photoelectron spectroscopy (XPS) was conducted on single crystals with Thermo ESCALAS 250 spectrometer, using the Mg *K*α (1253.6 eV) achromatic X-ray source. Thermogravimetric analyses (TGA) were recorded with a Setaram Themys HP thermal analyzer (Lyon, France) under a nitrogen flow with a temperature rate of 10 °C·min^−1^. The powder X-ray diffraction (PXRD) patterns were determined on a Rigaku X-Smartlab SE X-ray diffractometer (Tokyo, Japan) using Cu-Kα radiation (λ = 1.541 Å) to study the crystalline phase of the samples. UV-vis spectra were measured using a Shimadzu UV-3100 spectrophotometer (Kyoto, Japan) with DMSO as a solvent in room atmosphere. Photoluminescence (PL) properties were conducted on a Hitachi F-7000 fluorescence analyzer (Kyoto, Japan) in the range of 450–700 nm.

### 3.2. Syntheses

#### 3.2.1. Synthesis of {([Cu^II^(2,2′-bpy)_2_]_2_[PMo^VI^_8_V^V^_2_V^IV^_2_O_40_(V^IV^O)_2_])[Cu^I^(2,2′-bpy)]}∙2H_2_O (**1**)

Compound **1** was synthesized under hydrothermal conditions and the mixture of Na_3_[P(Mo_3_O_10_)_4_]·*x*H_2_O (0.66 g, 0.34 mmol), V_2_O_5_ (0.15 g, 0.82 mmol), CuCl_2_·2H_2_O (0.14 g, 0.80 mmol), H_2_C_2_O_4_∙2H_2_O (0.4 g, 3.17 mmol), 2,2′-bpy (0.11 g, 0.70 mmol) and distilled water (18 mL) was dissolved with magnetic stirring for about 120 min at room temperature. Then, the pH of the mixed solution was adjusted to 4 by titration with 6 M HCl. After the stirred solution was located into a 30 mL Teflon-lined autoclave and placed in an oven with heating from 4 °C per minute to 180 °C for 3 days under autogenous pressure, then closed and cooled naturally to room temperature. Finally, the obtained precipitate was washed with deionized water and dried at room temperature to form black polyhedral crystals (the yield was 0.64 g, 43.90% based on Mo). Anal. Calcd. for C_50_H_44_Mo_8_N_10_O_44_PV_6_Cu_3_: C, 21.60; H, 1.59; N, 5.03; Cu, 6.86; Mo, 27.60; V, 10.99; P, 1.01%. Found: C, 21.52; H, 1.61; N, 5.02; Cu, 6.93; Mo, 27.49; V, 10.91; P, 0.99%.

#### 3.2.2. Synthesis of {[Cu(2,2′-bpy)_2_]_2_[SiMo^VI^_8.5_Mo^V^_2.5_V^IV^_3_O_42_]}[Cu_0.5_(2,2′-bpy)(H_2_O)_0.5_] (**2**)

Compound **2** was prepared under hydrothermal conditions. Na_2_MoO_4_·2H_2_O (0.89 g, 3.68 mmol), V_2_O_5_ (0.15 g, 0.82 mmol), Na_2_SiO_3_·9H_2_O (0.098 g, 0.35 mmol), CuCl_2_·2H_2_O (0.14 g, 0.80 mmol), H_2_C_2_O_4_·2H_2_O (0.50 g, 3.97 mmol), 2,2′-bpy (0.125 g, 0.80 mmol) were added to 18 mL deionized water and dissolved with magnetic stirring for about 120 min at room temperature. Then, the pH of the mixed solution was adjusted to 4 by titration with 6 M HCl. After, the stirred solution was moved into a 30 mL Teflon-lined autoclave and placed in an oven with heating from 4 °C per minute to 180 °C for 3 days under autogenous pressure, then closed and cooled naturally to room temperature. Finally, the obtained precipitate was washed with deionized water and dried at room temperature to form black polyhedral crystals (the yield was 0.66 g, 71.25% based on Mo). Anal. Calcd. for C_45_H_36_Mo_11_N_9_O_42_SiV_3_Cu_2.5_: C, 19.45; H, 1.34; N, 4.54; Cu, 5.72; Mo, 37.98; V, 5.50; Si, 1.01%. Found: C, 19.33; H, 1.32; N, 4.51; Cu, 5.72; Mo, 37.91; V, 5.28; Si, 1.03%.

### 3.3. X-ray Crystallography Data Collection and Refinement Study 

The crystallographic data of compounds **1** and **2** were determined by a Bruker SMART CCD x-ray diffraction in *ψ-ω* scanning mode with graphite monochromated Mo-*K*α radiation (λ = 0.71073 Å) at normal temperature. No sign of crystal decay during single crystal data collections. Data restoration was accomplished by the SAINT procedure, and direct methods were used to solve the crystal structure by SHELXTL-2018/3 and the full-matrix least-squares method on *F*^2^ was used for crystal refinement and correction via SHELXTL-2018/3 crystallographic software package [72,73]. All atoms were corrected for anisotropy except Cu_2_ atom, O_24_, N_5_ and C_21_–C_25_ in the half a [Cu_0.5_(2,2′-bpy)(H_2_O)_0.5_]^+^ cation in **2**. The majority of hydrogen atoms were included in their geometrically calculated positions, but the hydrogen atoms of water molecular and disordered 2,2′-bpy in **1** and half 2,2′-bpy in **2** were not added. Supplementary crystal data and more detailed information for compounds **1** and **2** were stored at the Cambridge Crystal Data Center, and the CCDC numbers re 2,221,021 and 2,221,022. A summary of the crystal data and structure refinements for **1** and **2** is given in Table 3. 

## 4. Conclusions

In this work, two new hybrids based on different bi-capped Keggin POMs and Cu-bpy cations containing both Cu^II^ and Cu^I^ complexes were synthesized and characterized. The most striking feature of **1** is the (H_2_O)_2_ water cluster linking 1-D chains to 2-D supramolecular layer, and **2** contains a rare half-occupancy bi-coordinated [Cu_0.5_(2,2′-bpy)]^0.5+^ cation. Compounds **1** and **2** both show excellent catalytic activities for the styrene epoxidation to styrene oxide with aqueous TBHP and honorable photocatalytic degradation properties for RhB, MB and MB + RhB mixed solutions. The excellent catalytic activities of compounds **1** and **2** also prove that the modification of classical metal oxygen clusters by introducing appropriate transition metal ions is one of the effective means to obtaining high-activity cluster catalytic materials.

## Figures and Tables

**Table 1:** Hydrogen bond parameters for compounds **1** and **2**.

	D–H…A (Å)		D–H…A (Å)
Compound **1**			
Ow1…Ow1(#1)	2.705(2)	Ow1…O13	2.849(2)
Ow1…O8(#1)	3.003(2)	Ow1…O20(#2)	2.906(2)
Ow1…O6(#2)	3.259(2)	Ow1…O9(#1)	3.337(2)
Ow1…O9	3.119(3)	C4-H…O11(#3)	3.282(3)
C7-H…O18(#3)	3.2 75(2)	C7-H…O20(#3)	3.310(2)
C10-H…O21	3.081(2)	C10-H…O23	3.034(2)
C13-H…O10(#4)	3.301(3)	C17-H…O21(#5)	3.257(2)
N5-H…O12	3.384(2)	C5-H…O18	3.156(3)
Compound **2**			
C1-H…O8(#1)	3.276(8)	C11-H…O17	3.261(7)
C1-H…O23(#2)	3.365(6)	C12-H…O22	3.371(6)
C3-H…O9(#3)	3.096(7)	C13-H…O21(#2)	3.260(5)
C4-H…O9(#3)	3.302(9)	C16-H…O14	3.219(5)
C7-H…O8(#4)	3.338(6)	C17-H…O7(#6)	3.238(5)
C8-H…O10(#5)	3.113(8)	C18-H…O9(#7)	3.283(6)

Symmetric components for **1** #1: −*x*, −*y*, 2 − *z*; #2: −1 + *x*, *y*, *z*; 3#: 2 − *x*, −1 − *y*, 2 − *z*; #4: *x*, *y*, 1 + *z*; #5:1 − *x*, −1 − *y*, 3 − *z*, Symmetric components for **2** #1: 0.5 − *x*, −0.5 + *y*, 1.5 − *z*; #2: *x*, −*y*, 0.5 + *z*; #3: 1 − *x*, −*y*, 1 − *z*; #4: *x*, −*y*, −0.5 + *z*; #5: 1 − *x*, −*y*, 1 − *z*; #6: −*x*, −*y*, 1 − *z*; #7: −0.5 + *x*, −0.5 + *y*, *z.*

**Table 2:** Recyclabilities and reusabilities of compounds **1** and **2**.

Catalysts	Styrene Conversion (%)	Product Selectivity (%)		
		So	Bzdh	Others
Compound **1**				
Cycle 1	93.0	72.3	27.7	0
Cycle 2	90.5	60.8	39.0	0.86
Cycle 3	92.6	75.1	24.6	0
Compound **2**				
Cycle 1	87.3	91.1	8.9	0
Cycle 2	84.9	85.8	12.5	1.6
Cycle 3	83.8	91.5	8.5	0

So = styrene oxide; Bzdh = benzaldehyde; Others: including benzoic acid and phenylacetaldehyde.

**Table 3:** Crystal data and structure refinements for compounds **1** and **2**.

Head 1	Compound 1	Compound 2
Empirical formula	C_50_H_44_Mo_8_N_10_O_44_PV_6_Cu_3_	C_45_H_37_Mo_11_N_9_O_42.5_SiV_3_Cu_2.5_
Formula weight	2780.90	2779.04
Crystal system	triclinic	monoclinic
space group	*P*-1	*C*2/c
*a* (Å)	11.8892(11)	19.496(4)
*b* (Å)	12.8839(12)	22.677(5)
*c* (Å)	13.1485(13)	17.002(3)
α (^o^)	87.2930(10)	90
β (^o^)	81.7470(10)	95.28(3)
γ (^o^)	72.7150(10)	90
Volume (Å^3^)	1903.2(3)	7485(3)
*Z*	1	2
Crystal size (mm)	0.22 × 0.20 × 0.19	0.23 × 0.18 × 0.17
*D*_c_ (mg·m^3^)	2.429	1.231
*μ* (mm^−1^)	2.910	1.469
Completeness to *θ*	98.3	99.7
*θ* for data collection	2.06–27.20	3.00–27.48
*F*(000)	1341	2645
Reflection collected	11,377	35,774
Unique data (*R_int_*)	8194(0.0166)	8567(0.0200)
GOF on *F*^2^	1.022	1.079
parameters	592	520
*R* indices [*I > 2θ(I)*]	*R*_1_ ^a^ = 0.0494, ω*R*_2_ ^b^ = 0.1258	*R*_1_ ^a^ = 0.0545, ω*R*_2_ ^b^ = 0.1478
*R* indices (all data)	*R*_1_ ^a^ *=* 0.0686, ω*R*_2_ ^b^ = 0.1384	*R*_1_ ^a^ = 0.0585, ω*R*_2_ ^b^ = 0.1510
Δρ_max_/Δρ_min_, e Å^−3^	2.243 and −1.217	2.204 and −1.609

^a^ *R*_1_ = ∑||*F*_o_| − |*F*_e_||∑|*F*_o_|; ^b^ ω*R* = {[∑ω(*F*_o_^2^ − *F*_e_^2^)^2^][∑ω(*F*_o_^2^)^2^]}^1/2^.

## Data Availability

Date is available from the corresponding author.

## Supplementary Materials

The following supporting information can be downloaded at: https://www.mdpi.com/article/10.3390/molecules28124706/s1, Figures S1–S14 and Tables S1 and S2. References [51,52,68,69,70] are cited in the supplementary materials.

## Sample Availability

Samples of the compounds are not available from the authors.
