# Supplementary material for: Two New Compounds Based on Bi-Capped Keggin Polyoxoanions and Cu-Bpy Cations Contain Both CuII and CuI Complexes: Synthesis, Characterization and Properties
Source: Molecules. 2023 Jun 12;28(12):4706. doi: 10.3390/molecules28124706 (PMC10302035; doi:10.3390/molecules28124706)
Supplement: Supplementary file 1 [file molecules-28-04706-s001.zip › molecules-2425791-supplementary.pdf]

molecules

Supporting information

Two New Compounds Based on Bi-Capped Keggin Polyoxoanions and Cu-Bpy Cations Contain Both Cu^{II} and Cu^{I} Complexes: Synthesis, Characterization and Properties

Yabing Liu [1,2,*], Wentong Zhao [2], Jijun Zheng [2], Huan Wang [1,2], Xiaobing Cui [3] and Yaodan Chi [1,*]

1 Key Laboratory for Comprehensive Energy Saving of Cold Regions Architecture of Ministry of Education, Jilin Jianzhu University, Changchun 130118, China; wanghuan@jlju.edu.cn
2 College of Material Science and Engineering, Jilin Jianzhu University, Changchun 130118, China
3 State Key Laboratory of Inorganic Synthesis and Preparative Chemistry, College of Chemistry, Jilin University, Changchun 130021, China; cuixb@mail.jlu.edu.cn
* Correspondence: liuyab@163.com (Y.L.); chiyaodan@jlju.edu.cn (Y.C.)

Contents

Figure S1. Polyhedral representation of the structure of $[PMo^{VI}_8V^{V}_2V^{IV}_2O_{40}(V^{IV}O)_2]^{5-}$ in compound **1**.3

Figure S2. The coordination modes are both of disorder 2,2'-bpy and Cu(2) cations in compound **1**. ...3

Figure S3. Infrared spectra of compounds **1** (a) and **2** (b). 4

Figure S4. The XPS spectra of molybdenums of compounds **1** (a) and **2** (b)**.**....5

Figure S5. The XPS spectra of vanadiums in compounds **1** (a) and **2** (b)**.** 6

Figure S6. The TG curves of compounds **1** and **2**. 7

Figure S7. The PXRD patterns of **1** (a) and **2** (b). 8

Figure S8. The UV-vis spectra for compounds **1** and **2**. 9

Figure S9. Solid state fluorescence spectra of 2,2'-bpy (a), compound **1** (b) and compound **2** (c). 10

Figure S10. The experimental and cycle 3 IR spectra of compounds 1(a) and 2(b). 11

Figure S11. The simulated, experimental and powder XRD patterns after three runs of repeated experiments of compounds **1** (a) and **2** (b). 12

Figure S12. UV-vis absorption spectra of the RhB solution in the presence of compound **1** under UV irradiation at pH = 1(a), 3(b) and 10(c), and changes in C_t/C_o plot of RhB solution *versus* reaction time in the presence of compound **1** (d) at different pH values. 13

Figure S13. UV-vis absorption spectra of the RhB solution in the presence of compound **2** under UV irradiation at pH = 1(a), 3(b) and 10(c), and changes in C_t/C_o plot of RhB solution *versus* reaction time in the presence of compound **2** (d) at different pH values. 14

Figure S14. Changes of concentration for MB and RhB+MB solution in the presence of compound **1** and compound **2** under different irradiation times at pH = 3. (a) **1**-MB, (b) **2**-MB, (c) **1**-RhB+MB and (d) **2**-RhB+MB. 15

Table S1. Catalytic activity and product distribution of compounds **1**, **2**, **4** and **5**. 16

Table S2. Comparison of the degradation rate of RhB dyes by various photocatalysts........ 17

References 17

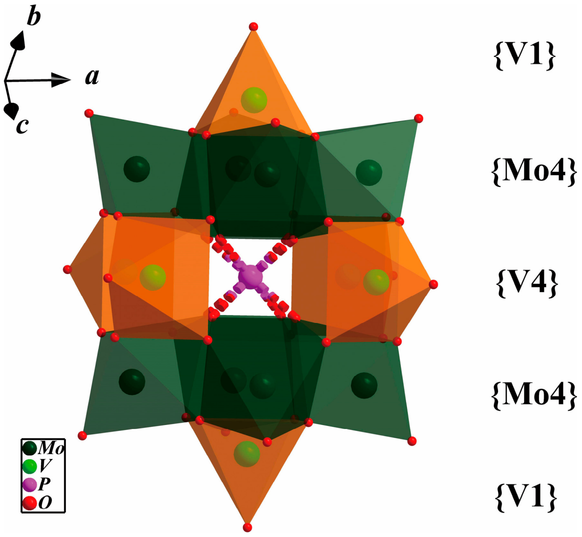

Figure S1. Polyhedral representation of the structure of $[PMo^{VI}_8V^{V}_2V^{IV}_2O_{40}(V^{IV}O)_2]^{5-}$ in compound **1**.

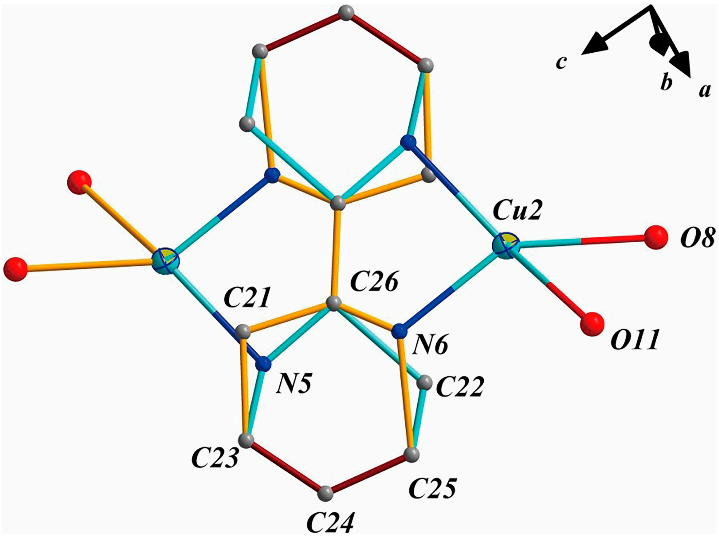

Figure S2. The coordination modes are both of disorder 2,2'-bpy and Cu(2) cations in compound **1**.

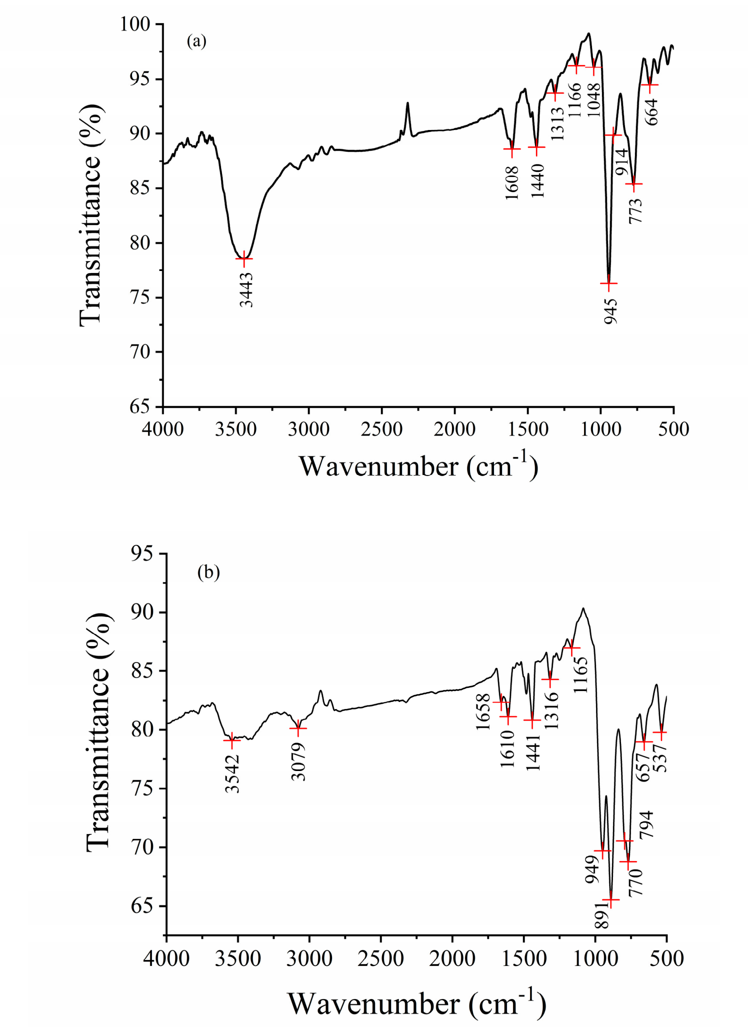

Figure S3. Infrared spectra of compounds **1** (a) and **2** (b).

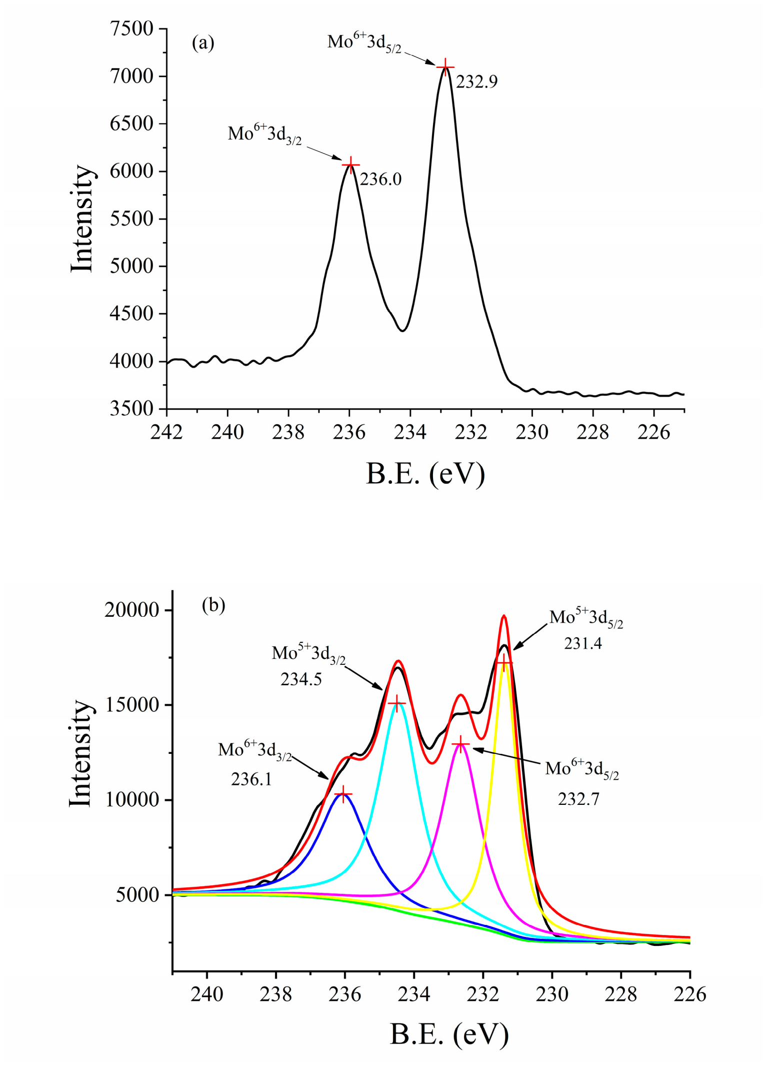

Figure S4. The XPS spectra of molybdenums of compounds **1** (a) and **2** (b)**.**

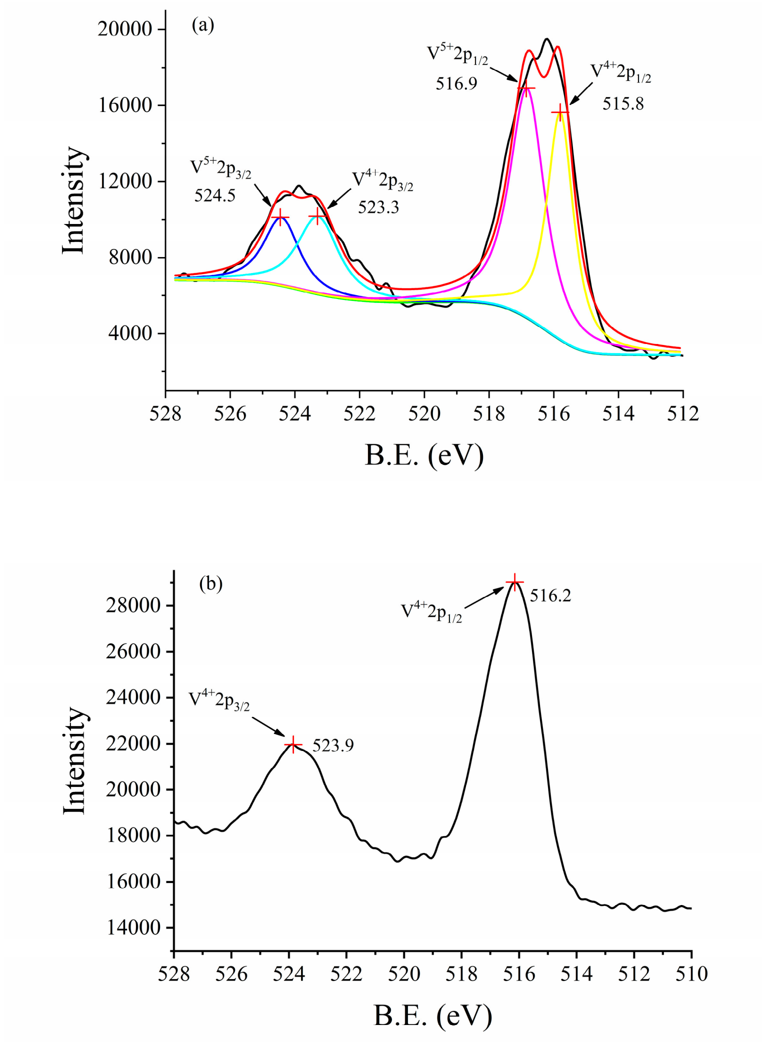

Figure S5. The XPS spectra of vanadiums in compounds **1** (a) and **2** (b)**.**

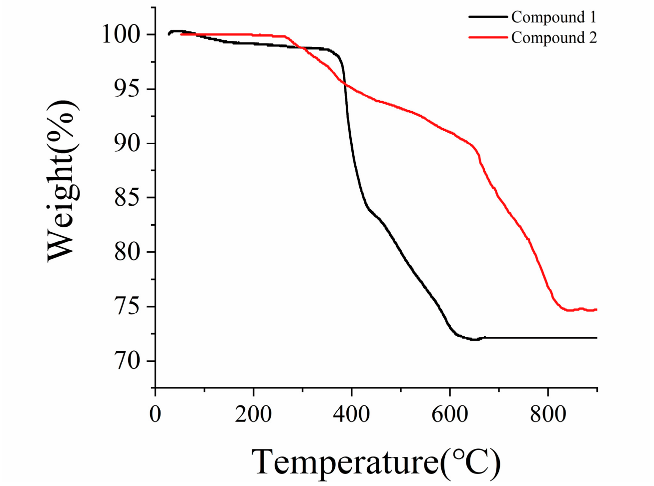

Figure S6. The TG curves of compounds **1** and **2**.

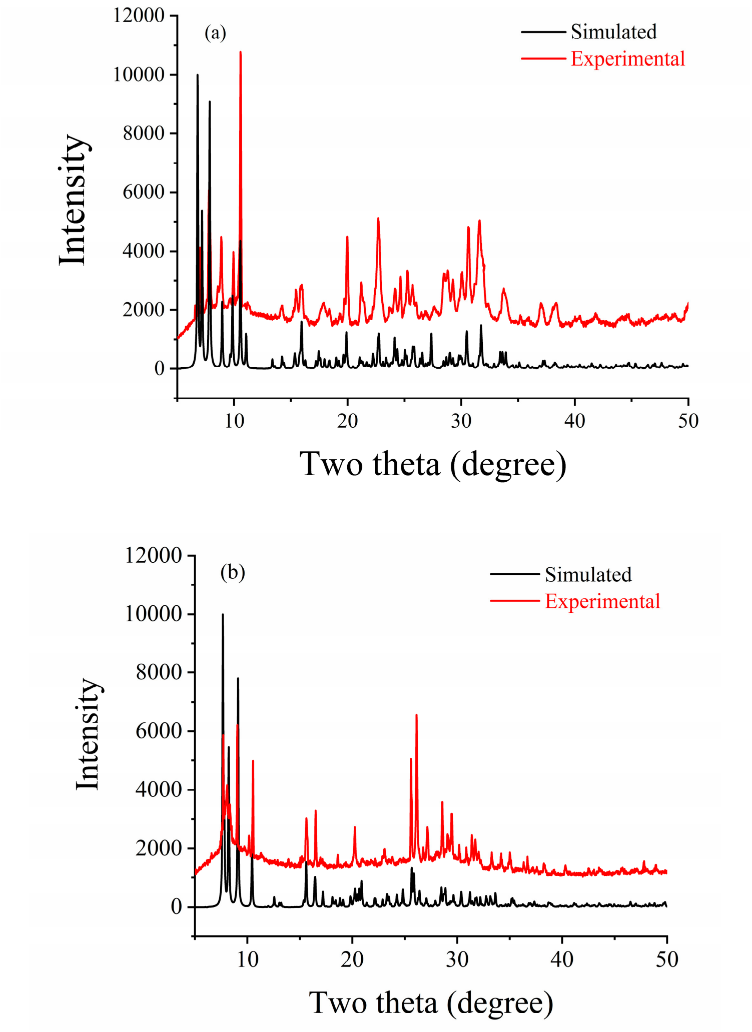

Figure S7. The PXRD patterns of **1** (a) and **2** (b).

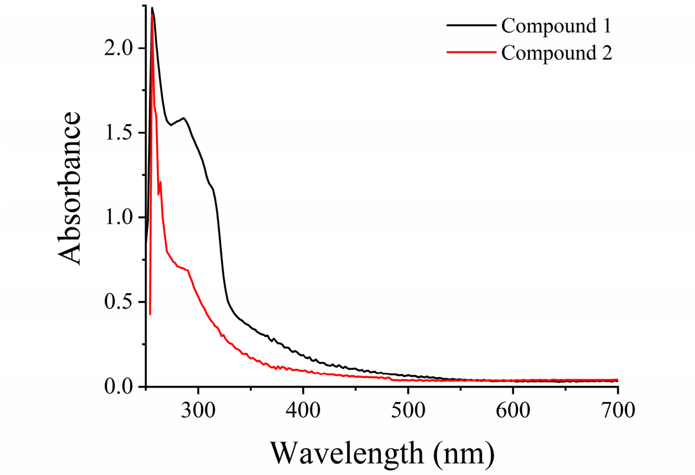

Figure S8. The UV-vis spectra for compounds **1** and **2**.

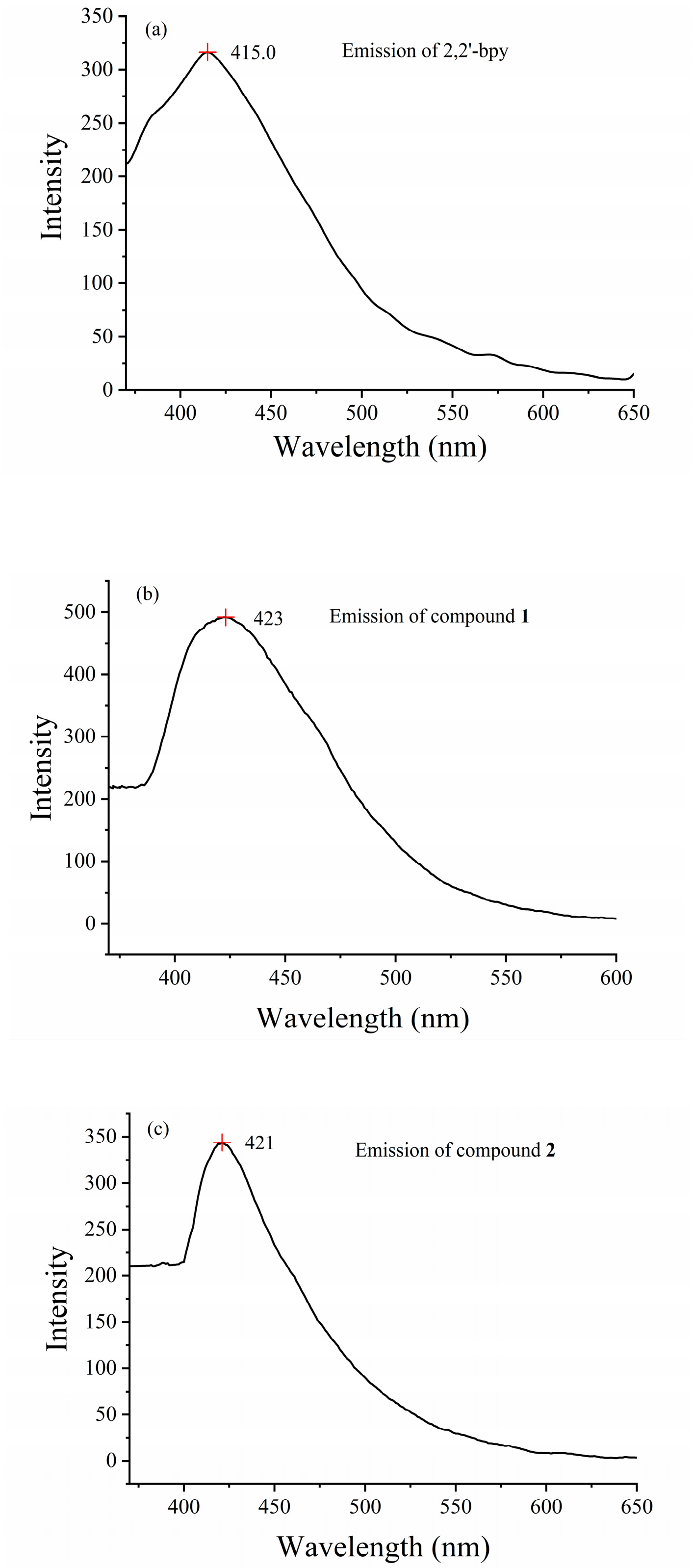

Figure S9. Solid state fluorescence spectra of 2,2’-bpy (a), compound **1** (b) and compound **2** (c).

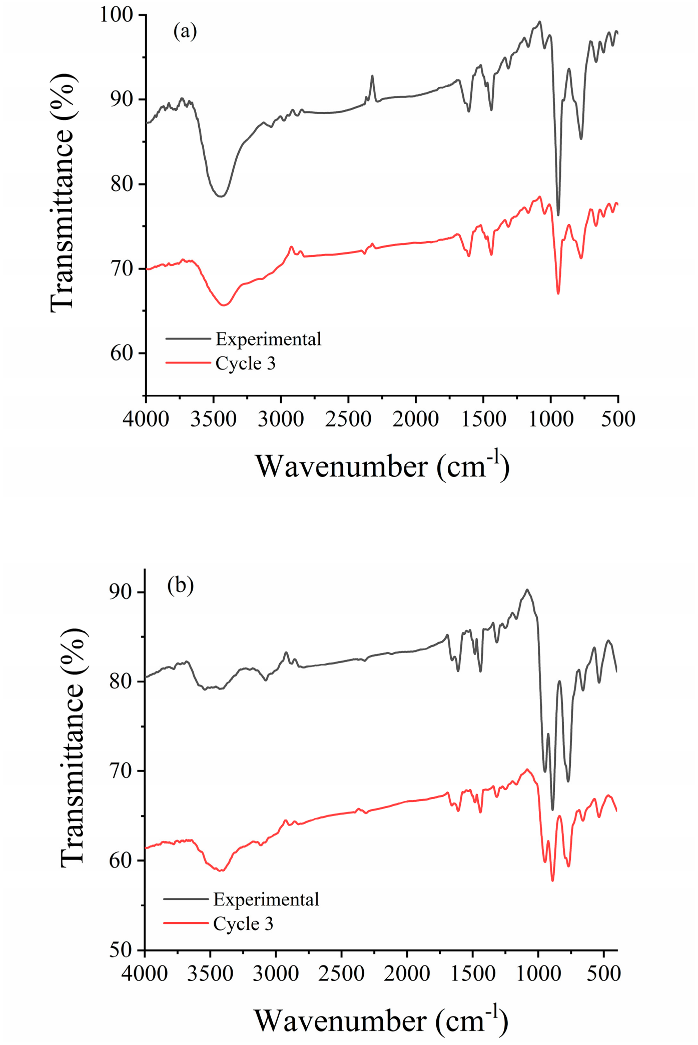

Figure S10. The experimental and cycle 3 IR spectra of compounds 1(a) and 2(b).

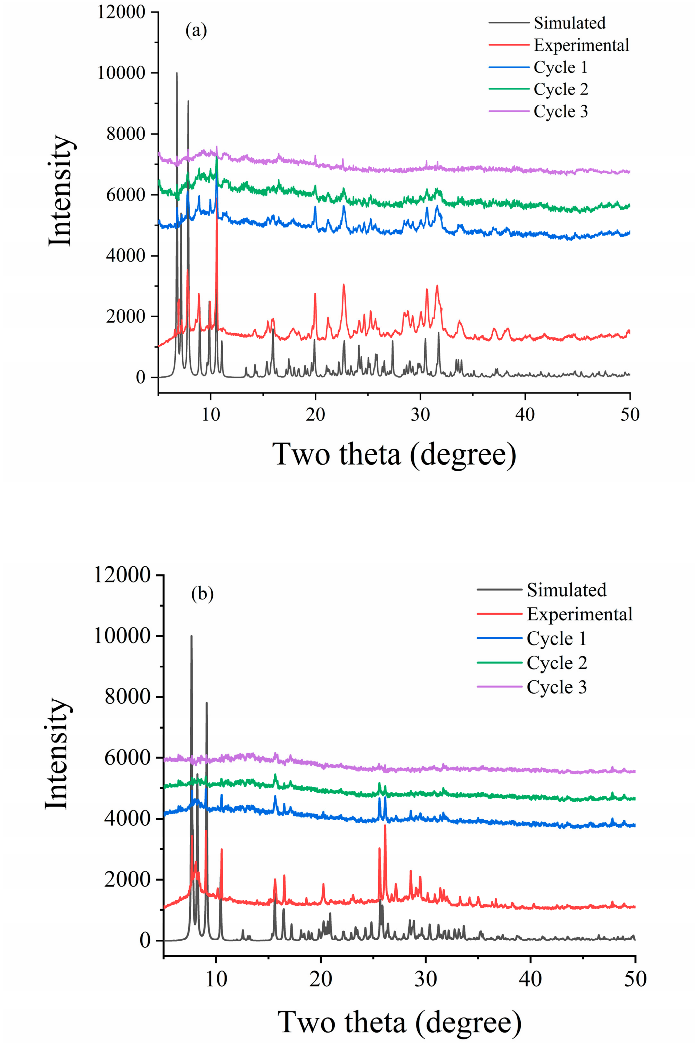

Figure S11. The simulated, experimental and powder XRD patterns after three runs of repeated experiments of compounds **1** (a) and **2** (b).

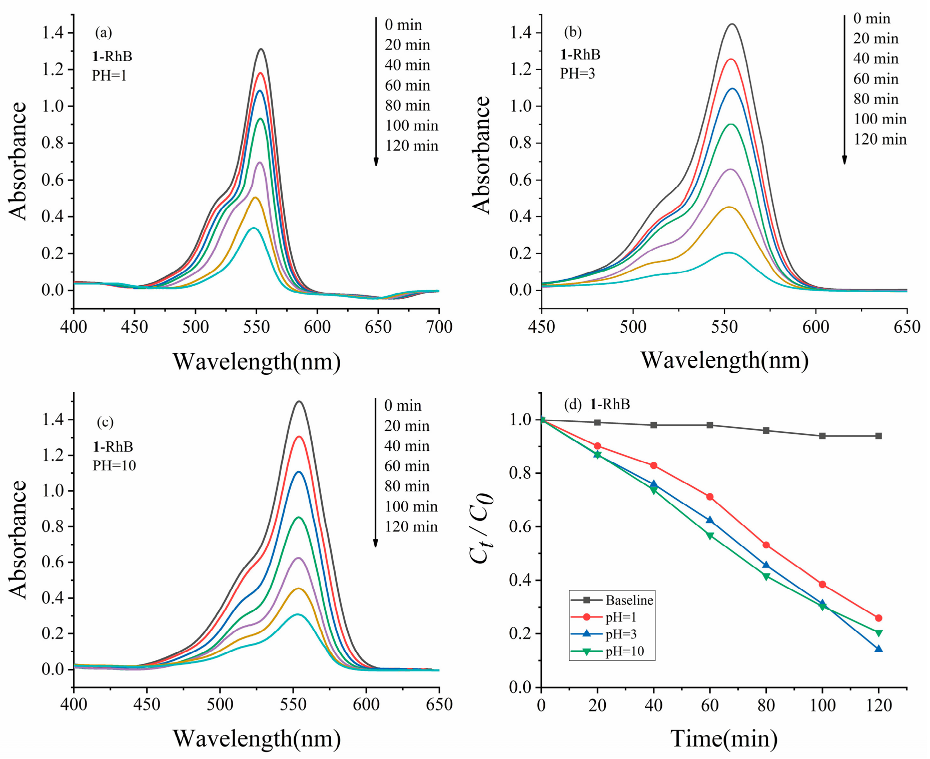

Figure S12. UV-vis absorption spectra of the RhB solution in the presence of compound **1** under UV irradiation at pH = 1(a), 3(b) and 10(c), and changes in C_t/C_o plot of RhB solution *versus* reaction time in the presence of compound **1** (d) at different pH values.

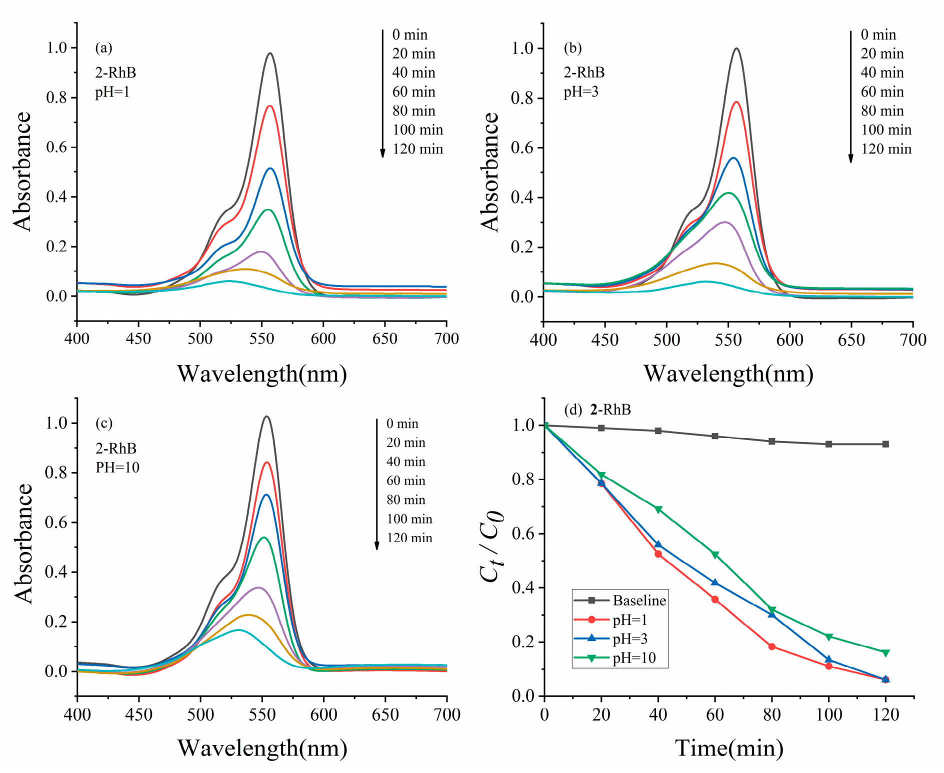

Figure S13. UV-vis absorption spectra of the RhB solution in the presence of compound **2** under UV irradiation at pH = 1(a), 3(b) and 10(c), and changes in C_t/C_o plot of RhB solution *versus* reaction time in the presence of compound **2** (d) at different pH values.

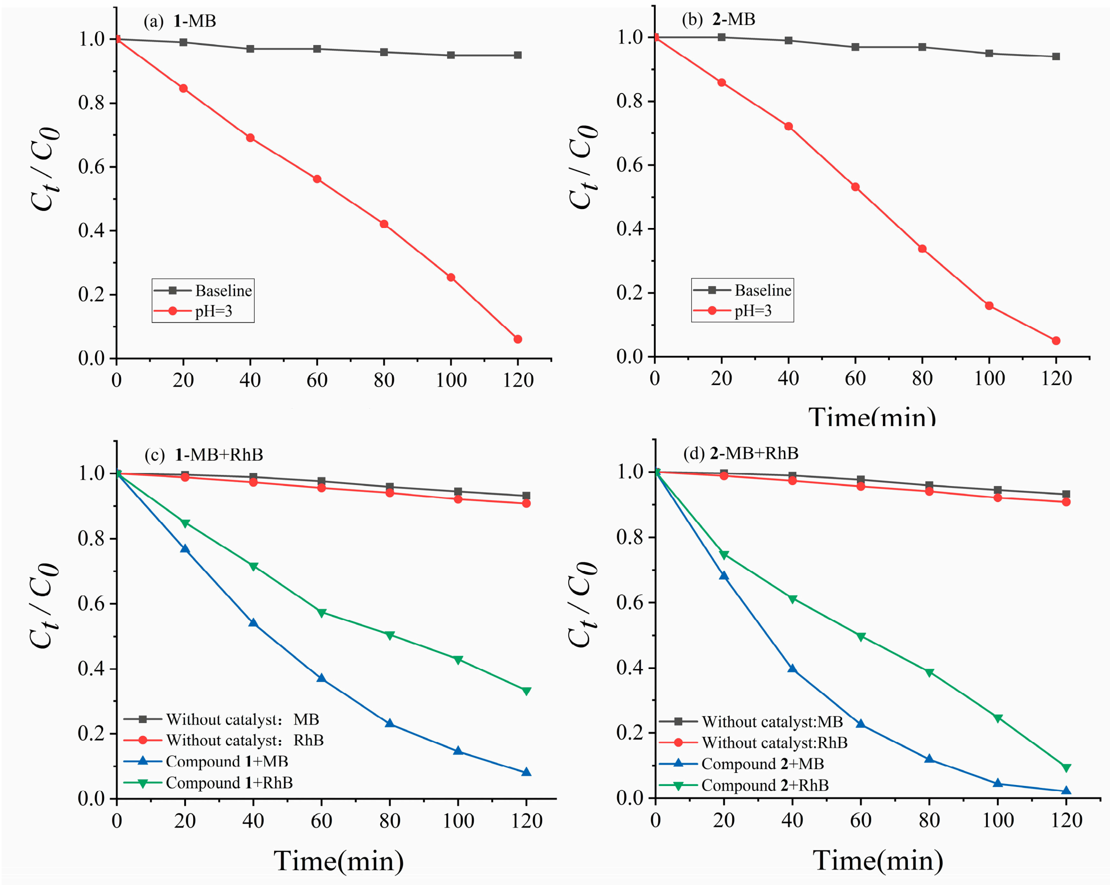

Figure S14. Changes of concentration for MB and RhB+MB solution in the presence of compound **1** and compound **2** under different irradiation times at pH = 3. (a) **1**-MB, (b) **2**-MB, (c) **1**-RhB+MB and (d) **2**-RhB+MB.

Table S1. Catalytic activity and product distribution of compounds **1**, **2**, **4** and **5**.

Catalysts	Styrene conversion (%)	Product selectivity (%)		
		**So**	**Bzdh**	**Others**
Compound **1**	91.7	65.9	34.09	0.0
Compound **2**	86.7	89.4	6.0	0.86
Compound **4** [51]	97.2	70.1	22.5	7.4
Compound **5** [52]	90.0	87.2	5.0	0.0

So = styrene oxide; Bzdh = benzaldehyde; Others: including benzoic acid and phenylacetaldehyde.

Table S2. Comparison of the degradation rate of RhB dyes by various photocatalysts.

Photocatalyst	Ligands	Time (min)	degradation (%)	Ref.
$(NBu_4)_4[SiW_{12}O_{40}]$ (**6**)	no	300	34.4	[70]
$[Cu_2(bipy)][H_2SiW_{12}O_{40}]\cdot(bib)\cdot 2H_2O$ (**7**)	bipy	300	51.3	[70]
$\{[Co_2(btp)_3(H_2O)_6](\alpha\text{-}SiW_{12}O_{40})\cdot 3H_2O\}_n$ (**8**)	btp	210	70.2	[71]
$[Zn(bix)_4][PMo_9V_3O_{40}(VO)_2]\cdot 2H_2O$ (**9**)	bix	210	88.1	[72]
$[HMn(bix)_4][PMo_8V_4O_{40}(VO)_2]\cdot 2H_2O$ (**10**)	bix	210	64.8	[72]
$[Cu(bix)_4][PMo_9V_3O_{40}(VO)_2]\cdot 4H_2O$ (**11**)	bix	210	80.0	[72]
$\{([Cu(2,2'\text{-}bpy)_2]_2[PMo_8V_4O_{40}(VO)_2])$ $[Cu(2,2'\text{-}bpy)]\}\cdot 2H_2O$ (**1**)	bpy	120	85.8	This work
$\{[Cu(2,2'\text{-}bpy)_2]_2[SiMo_{11}VO_{40}(VO)_2]$ $[Cu_{0.5}(2,2'\text{-}bpy)(H_2O)_{0.5}]$ (**2**)	bpy	120	94.0	This work

NBu_4= tert-Butylamine; bipy= 4,4'-bipyridine; dpb = 2,6-di(2-pyridine)-4,4'-bipyridine; bix=1,4-Bis(imidazole-1-ylmethyl)benzene); bpy= bipyridine.

References

[51] Xiao, L.N.; Zhang, H.; Zhang, T.T.; Zhang, X.; Cui, X.B. Two new POMOF compounds constructed from polyoxoanions, metals and organic ligands. *J. Solid State Chem.* **2018**, 259, 11-18. [CrossRef]

[52] Fu, L.V.; Guo, H.Y.; Hu, Y.Y.; Guo, L.L.; Yu, M.; Cui, X.B.; Xu, J.Q. Polyoxometalate tri-supported transition metal complexes containing mixed-valent transition metal ions. *J. Coord. Chem.* **2015**, 68, 3814-3824. [CrossRef]

[70] Li, L.; Sun, J.W.; Sha, J.Q.; Li, G.M.; Yan, P.F.; Wang, C. Construction of POMOFs with different degrees of interpenetration and the same topology. *CrystEngComm*. **2015**,*17*,846-854. [CrossRef]

[71] Wu, Y.Y.; Liu, Y.; Huang, Y.J.; Xiao, G.; Li, Y.M.; Bai, Y.Do; Dang, D.B. Five isomorphous polyoxometalate-based inorganic-organic hybrid materials comprising 2D metal-organic layers. *J. Solid State Chem.* **2020**, *286*, 121302. [CrossRef]

[72] Ding, Y.; Meng, J.X.; Chen, W.L.; Wang, E.B. Controllable assembly of four new POM-based supramolecular compounds by altering the POM secondary building units from pseudo-Keggin to classic Keggin. *CrystEngComm*. **2011**, *13*, 2687-2692. [CrossRef]
